# Urban–Rural Disparities in the Lung Cancer Surgical Treatment Pathway: The Paradox of a Rich, Small Region

**DOI:** 10.3389/fsurg.2022.884048

**Published:** 2022-04-28

**Authors:** Eleonora Maddalena Minerva, Adele Tessitore, Stefano Cafarotti, Miriam Patella

**Affiliations:** Thoracic Surgery Department, Ospedale Regionale di Bellinzona e Valli, Via Ospedale, Bellinzona, Switzerland

**Keywords:** lung cancer, treatment disparities, surgical treatment, rurality, screening

## Abstract

**Introduction:**

Rural populations in large countries often receive delayed or less effective diagnosis and treatment for lung cancer. Differences are related to population-based factors such as lower pro capita income or increased risk factors or to differences in access to facilities. Switzerland is a small, rich country with peculiar geographic and urban characteristics.

We explored the relationship between lung cancer diagnostic–surgical pathway and urban–rural residency in our region.

**Methods:**

We retrospectively analyzed the medical records of 280 consecutive patients treated for primary non-small cell lung cancer at our institution (2017–2021). This is a regional tertiary center for diagnosis and treatment, and data were extracted from a prospectively collected clinical database. We included anatomical lung resection. Collected variables included patients and surgical characteristics, risk factors, comorbidities, histology and staging, symptoms (vs. incidental diagnosis), general practitioner (GP) involvement, health insurance, and suspected test-treatment interval. The exposure was rurality, defined by the 2009 rural–urban residency classification from the Department of Land.

**Results:**

A total of 150 patients (54%) lived in rural areas. Rural patients had a higher rate of smoking history (93% vs. 82%; *p* = 0.007). Symptomatic vs. incidental diagnosis did not differ as well as previous cancer rate, insurance, and pathological staging. In rural patients, there was a greater burden of comorbidities (mean Charlson Comorbidity Index Age-Adjusted 5.3 in rural population vs. 4.8 in urban population, *p* = 0.05), and GP was more involved in the diagnostic pathway (51% vs. 39%, *p* = 0.04). The interval between the first suspected test and treatment was significantly shorter (56 vs. 66.5 days, *p* = 0.03). Multiple linear regression with backward elimination was run. These variables statistically predicted the time from the first suspected test and surgical treatment [*F*(3, 270), *p* < .05, *R*^2^ = 0.24]: rurality (*p* = 0.04), GP involvement (*p* = 0.04), and presence of lung cancer-related symptoms (*p* = 0.02).

**Conclusions:**

In our territory with inhomogeneous population distribution and geographic barriers, residency has an impact on the lung cancer pathway. It seems paradoxical that rural patients had a shorter route. The more constant involvement of GP might explain this finding, having suggested more tests for high-risk patients in the absence of symptoms or follow-ups. This did not change the staging of surgical patients, but it might be essential for the organization of an effective lung cancer screening program.

## Introduction

In the recent past, there has been increasing interest in the literature in studying cancer outcome inequalities among population subgroups, such as urban vs. rural residents. Rurality is often presented as a risk factor for poor cancer outcomes in various types of malignancies (1–3).

The causes of these rural–urban disparities in lung cancer prognosis are yet poorly defined (4). It seems that people living in the countryside have more risk factors than urban residents. Several studies have recognized among rural residents higher rates of cancer-risk behaviors such as cigarette smoking, poorer diet, alcohol abuse, physical inactivity, and obesity (4–6). An additional explanatory factor in broad countries such as the United States and Australia is that a considerable percentage of uninsured or underinsured patients come from rural regions (1, 4, 6–8). Furthermore, rural inhabitants often present lower incomes and lower educational levels than their urban counterparts (1, 2, 6, 7). The result of these factors combined is a delay in healthcare referral. This delay causes in turn a later disease stage at presentation, meaning a reduced range of therapeutic choices and worse survival rates (1, 4).

However, the above-mentioned data are mostly extracted by studies performed in extremely wide and non-homogeneous countries, in terms of both population and socio-economical levels, such as the United States and Australia (1–3). Therefore, it seems to be interesting to study and verify this phenomenon at a local level in order not to generalize the results (8, 9), especially with a view to introducing a screening program (10). Switzerland is a tiny state with 8,670,000 inhabitants, with one of the highest per capita incomes worldwide. Its great environmental and administrative diversity may render the Helvetic Confederation a case study for the peculiar distribution of its healthcare system. Canton Ticino is not an exception, and the Department of Land provides a detailed chart of this variability, defining “functional regions” based on the local socio-economical development (11, 12).

Our interest as oncological surgeons of the regional referral center is to analyze timing and possible variability of the diagnostic–therapeutic pathway of lung carcinoma within our region.

## Patients and Methods

### Territorial and Population Setting

Tessin is a region defined as a canton in Switzerland, with a population of 351,000 residents. Its area extends 2,812.21 km^2^, with 80% of the land being constituted by mountains and 15% by hills. Approximately half of the inhabitants live in urban areas, while the rest of the population is distributed in rural territories (11). Based on population density, residency areas are divided into five categories according to the description provided by the Department of Land (12). High-density area (>80 inhabitants per hectare) is defined as primarily urban. This is followed by the communities of the suburban area (45–80 inhabitants per hectare), with decreasing density and specific urban characteristics. These two areas make up the agglomeration (urban), while the periurban, the hinterland, and the mountains constitute the suburban rural areas (<45 inhabitants per hectare).

The Swiss healthcare system is based on a federalist structure, which means that the federal government, cantons, and local municipalities assume different tasks in the healthcare system. In accordance with the Swiss Federal Health Insurance Act (KVG/HIA), basic insurance is compulsory for anyone and it can be public, semi-private, or private, depending on the level of coverage.

According to the Euro Health Consumer Index Report (13), which took into consideration patient rights, access to healthcare, preventive care, and a range of benefits, Switzerland ranked first in 2018.

### Lung Cancer Pathway

Ente Ospedaliero Cantonale is the public regional tertiary center for the diagnosis and treatment of lung cancer with specific facilities and surgical expertise (14) and multidisciplinary team (MDT) discussion on a regular basis.

Patients with suspected/ascertained lung cancer are referred for MDT discussion and selected for surgery according to current functional (15) and oncological (16) guidelines. Referring physicians can include internal specialists as well as general practitioners (GPs) and other external physicians from either public or private settings.

### Eligibility and Data Collection

The database containing the consecutive anatomical lung resections (segmentectomy, lobectomy, bilobectomy, and pneumonectomy) performed at our institution was screened. Being a clinical prospectively collected database, the completeness and the accuracy of the data were extremely high, with the maximum rate of missing data being 3% for a single variable. Between January 2017 and December 2021, the database included 350 procedures. Patients with a diagnosis of primary non-small cell lung cancer (NSCLC) were included in the study. Patients with a diagnosis of small cell lung cancer, typical carcinoid, benign disease, metastasis from other organs, and recurrence of lung cancer were excluded. For the purpose of the study, we also excluded patients who underwent neoadjuvant treatments. The study was approved by the local ethical committee (protocol number 2022-00184).

Collected variables included patients and surgical characteristics, together with risk factors (smoking exposure) and comorbidities [Charlson Comorbidity Index Age-Adjusted (CCI)], histology, symptoms (vs. incidental diagnosis), GP involvement, and type of health insurance. The outcome of interest was the suspected test-treatment interval (defined as the number of days between the first imaging leading to a suspicion of lung cancer and the surgical treatment). To verify the possible impact on cancer prognosis, we also analyzed the final pathological staging. The exposure variable was rurality.

Screened variables were considered based on their impact on the rapidity of the diagnostic–therapeutic pathway. As we can find in the literature, we identified possible confounders that could contribute to making a variation in the lung cancer diagnostic pathway: previous history of malignancy requiring follow-up, presence of lung cancer-related symptoms, and insurance status (5, 17, 18). Patients affected by previous cancer undergo radiological follow-ups, during which incidental findings of lung cancer can be observed with a higher probability compared to patients who do not undergo regular imaging (17). Moreover, the presence of typical symptoms usually leads to a timely diagnosis rather than their absence (18). Eventually, underinsured people are less likely to seek early medical attention in order not to face supplementary expenses (5).

### Statistical Analysis

Collected variables and outcomes were analyzed and compared in the two groups of exposure. Continuous variables were tested for normality with the Shapiro–Wilk *W* test. Normally distributed variables were analyzed using the Student *t*-test; skewed distributed ones were analyzed with the Mann–Whitney *U* test. Categorical variables were tested by means of Fisher’s exact test (in case the number of observations was less than 10 in at least one cell) or the chi-squared test. Variables with statistical significance difference at univariable analysis and, regardless of their statistical significance, variables considered as potential confounders were included in a multiple regression analysis with backward elimination to verify the impact on the outcome. The level of significance was set at 0.05; all statistical analyses were performed using STATA software (StataCorp LLC, College Station, TX, USA).

## Results

**Figure 1:**
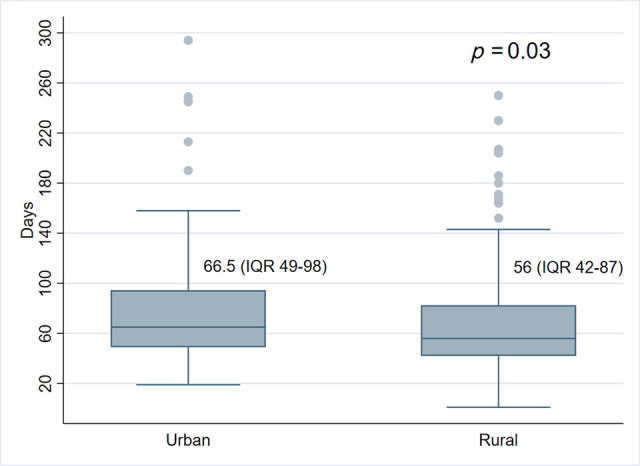
Median first suspected test-operation interval in urban–rural patients.

A total of 280 patients were eligible for the analysis. A total of 150 patients (54%) lived in rural areas. We performed 248 lobectomies/bilobectomies, 28 segmentectomies, and 4 pneumonectomies (**Table 1**). Lymphadenectomy was performed in all cases. Demographic characteristics did not differ significantly across residencies. Rural patients had a higher rate of smoking history (93% vs. 82%; *p* = 0.007) and a slightly significant trend to have a greater burden of comorbidities (mean CCI 4.8 vs. 5.3, *p* = 0.05). The number of patients who had positron emission tomography scan, preoperative mediastinal staging by means of endoscopic ultrasound or mediastinoscopy, and the number of cancer diagnoses obtained before the curative resection did not differ between groups. For rural patients, GPs were significantly more involved in the diagnostic pathway (51% vs. 39%, *p* = 0.04) and the interval between the first suspected test and treatment was significantly shorter (56 vs. 66.5 days, *p* = 0.03). The cumulative median interval time between the first suspected test and surgery was 61 days (IQR: 46–90) with a difference of 10.5 days. **Figure 1** shows the difference in days between the median interval between the first suspected test and treatment for the two groups. The final pathological staging did not differ in the two groups. Adenocarcinoma histology was more frequent in urban patients (78% vs. 67%, *p* = 0.04).

**Table 1:** Patients’ characteristics and differences based on the results of univariable analysis.

Variables	Urban	Rural	Total	*p*-Value
Population	130 (46)	150 (54)	280	
Lobectomy/bilobectomy	113	135	248	0.6
Segmentectomy	14	14	28	
Pneumonectomy	3	1	4	
Gender, male	70 (54)	83 (55)	153 (54.6)	0.8
Age (years) (mean, SD)	70.7 ± 8.6	69.6 ± 8.8	70 ± 8.7	0.6
Smoking history	107 (82)	140 (93)	247 (88)	0.007
Body mass index (mean, SD)	25.5 (5.1)	25.8 (4.5)	25.7 (4.8)	0.2
FEV1% (mean, SD)	87 (21.6)	84 (18.9)	85 (20.2)	0.2
DLCO (mean, SD)	73 (19.7)	72 (19)	72.7 (19.3)	0.5
Charlson comorbidity Index age-Adjusted (mean, SD)	4.8 (1.8)	5.3 (2.2)	5 (2)	0.05
Performance status 0	87 (67)	100 (66)	187 (67)	0.9
Private/semi-private health insurance	31 (24)	42 (28)	73 (26)	0.4
General physician involvement	51 (39)	77 (51)	128 (46)	0.04
Lung cancer symptoms	29 (22)	31 (21)	60 (21.4)	0.6
Previous cancer	36 (28)	48 (32)	84 (30)	0.4
Preoperative invasive mediastinal staging	41 (31)	53 (35)	94 (33.6)	0.5
Diagnosis before resection	69 (53)	84 (56)	153 (54.6)	0.6
First test-operation interval (days) (median, IQR)	66.5 (49–98)	56 (42–87)	61 (46–90)	0.03
Adenocarcinoma histology	101 (78)	100 (67)	201 (71.7)	0.04
Squamous cell carcinoma histology	21 (16)	37 (24)	58 (20.8)	0.08
Other histology	8 (6)	13 (9)	21 (7.5)	0.07
Pathological staging ≥IIA	46 (35)	57 (38)	103 (36.8)	0.6

*SD, standard deviation; FEV1, forced expiratory volume in 1 s; DLCO, diffusing capacity for carbon monoxide; IQR, interquartile range.*

*Results are expressed as numbers (percentage) unless otherwise specified.*

The number of symptomatic vs. incidental diagnoses did not differ between groups (**Table 2**). Among symptomatic patients, there was no difference in the interval between the first suspected test and treatment [median 57 days (IQR: 46–91) vs. 56 days (IQR: 40–73); *p* = 0.2]. Neither was there a statistical difference in the interval between symptoms onset and the first suspected test for the same patients [median 33.5 days (IQR: 12.5–73.5) vs. 29.5 days (IQR: 10–61); *p* = 0.8]. On the other hand, regarding patients with no cancer-related symptoms, we found a statistically significant difference in the interval between the first suspected test and treatment across urban/rural residency [median 69.5 days (IQR: 53–105) vs. 60.5 days (IQR: 42–92); *p* = 0.04]. The number of patients under regular follow-ups for previous malignancies was similar in the two groups, as well as the insurance status.

**Table 2:** Details of analysis according to the presence/absence of cancer-related symptoms.

Variables	Urban	Rural	Total	*p*-Value
Lung cancer symptoms (*n*, %)	29 (22)	31 (21)	60 (21.4)	0.6
First test-operation interval (days) (median, IQR)	57 (46–91)	56 (40–73)	54.5 (43.5–75)	0.2
Symptoms onset-first test (days) (median, IQR)	33.5 (12.5–73.5)	29.5 (10–61)	30.5 (10–62)	0.8
Incidental diagnosis (*n*, %)	98 (75.4)	118 (78.6)	216 (77.1)	0.6
First test-operation interval (days) (median, IQR)	69.5 (53–105)	60.5 (42–92)	63.5 (46–96.5)	0.04
History of symptoms not known (*n*, %)	3 (2.3)	1 (0.7)	4 (1.4)	0.7

*IQR, interquartile range.*

Results of the univariable analysis are summarized in **Table 1**.

Statistical significantly different variables and confounders were included in a multivariable analysis. Multiple linear regression with backward elimination was run. These variables statistically predicted the time from the first suspected test and surgical treatment [*F*(3, 270), *p* < 0.05, *R*^2^ = 0.24]: rurality (*p* = 0.04), GP involvement (*p* = 0.04), and presence of lung cancer-related symptoms (*p* = 0.02) (**Table 3**).

**Table 3:** Results of multiple linear regression analysis after backward elimination.

Variable	*B*	SE	*t*	*p*-Value	95% CI	*R* ^2^	*F*	Model *p*-Value
Rural residency	−20.6	8.3	−1.18	0.04	−26.2 −6.5	0.24	3.27	0.04
GP involvement	17.2	8.6	2.0	0.04	1.27 34.2			
Lung cancer symptoms	−9.8	10.3	−2.28	0.02	−44 −3.3			

*GP, general practitioner; B, unadjusted coefficient; SE, standard error; CI, confidence interval.*

## Discussion

Our analysis showed that patients residing in rural areas in Tessin had a shorter lung cancer diagnostic and surgical treatment pathway compared with the ones who lived in urban areas, with a difference of 10.5 days between groups. These results are in contrast with most published literature on this topic. As demonstrated by the large number of papers published in the last few years, the interest in the disparities in cancers incidence, timing of diagnosis, appropriateness of treatment, and survival, related to patients’ residency, is constantly increasing (1–6). Most studies showed that all indicators have a steady inverse relationship with residency as they progressively worsened with increasing remoteness from the urban area (2). The reason leading to these disparities could be found in population-based factors or in provider issues. Increased rural lung cancer mortality might be explained by increased smoking prevalence in rural populations with subsequent greater cancer incidence (2). In general, the major behavioral determinants of cancer, such as smoking, diet, alcohol use, and occupational and environmental exposures, are influenced by the individual- and area-socio-economic factors (19).

In our population, we identified higher smoking exposure prevalence in rural populations even though this did not strictly correspond with socio-economic differences, as indirectly demonstrated by the lack of association with the insurance level. Indeed, we should consider that talking about economic deprivation might not be appropriate, as Switzerland is one of the richest European Countries. In 2018, the GDP (gross domestic product) percapita in Tessin stood at 94,700 US dollars, which is well above the average of the Western European Countries and the United States (34,000 and 63,600 US dollars, respectively) (19–21).

To make a constructive comparison with the published literature, we should also underline that most studies have been conducted in large countries as the United States and Australia (1–6), in which the population density and the extent of the geographic area are extremely different compared to a Swiss region. However, Tessin has its peculiar territorial organization and geographic characteristics. From the administrative point of view, residential areas are fragmented in more than 100 municipalities, and the territory is 80% mountainous. Moreover, if we consider historical and cultural aspects, Tessin is characterized by strong and ingrained parochial behaviors toward foreign countries but also internally within the canton (22). Along with the multicenter organization of the public hospital (EOC) divided into four structures located in the four major cities, it certainly contributes to patients’ reluctance to move between different hospital facilities for specialist visits. It is therefore important to study the association between cancer pathways disparities and rurality at the local level. As demonstrated by other authors, rurality can increase or decrease cancer-related risks and outcomes, suggesting a complex pattern (8).

After multivariable analysis, and controlling for confounders, we confirmed that the elements influencing the time between the first suspected test and surgical treatment were rurality, GP involvement, and the presence of lung cancer-related symptoms. This refers to the early stages of NSCLC for which upfront surgery is indicated.

These results are particularly interesting in the light of a future lung cancer screening program. The main difference in the timing from the first suspected test and surgery between urban–rural residents has been found in the group of non-symptomatic patients. Awareness of lung cancer symptoms seems equal across urban–rural residency, which is not a given assumption, as demonstrated by studies on other types of cancer (1). While there was no difference in the rate of patients under follow-up for previous malignancies, we found a statistical difference in comorbidities burden between the urban–rural groups. This might mean that rural residents presented more risk factors for lung cancer and underwent more prompt screening examinations in the absence of lung cancer symptoms. Moreover, the GP involvement during the diagnostic process, which was greater in the rural group, might have played a role. GPs represent the frontline healthcare professional with a comprehensive knowledge of patients’ general status, behavior, and clinical history, and the relationship of trust should not be underestimated. Whereas rurality has been pointed out as a limit in lung cancer screening programs in other studies because of the lack of access to facilities (10) or inadequacy of referral (23), our results indirectly suggest that people living in rural areas in Tessin have an adequate healthcare support. Enhancing symptoms awareness is important in detecting lung cancer diagnosis, but it seems to be not sufficient to change prognosis and survival and to increase GP attendance (24). In line with our results, efficient GP to specialist referral is a key point to minimizing delays in lung cancer diagnostic and treatment pathways (25).

Our study has some limitations. We analyzed only the lung cancer diagnosis that underwent surgical treatment. This means that we focused only on a specific population with early stages, sufficient reserves to support lung resection, and on the treatment with curative intent. We did not analyze advanced stages, which represent most lung cancer diagnoses. Indeed, we can extrapolate some data from a report from the Ticino Cancer Registry: in 2015–2016, out of 367 diagnoses of NSCLC, 50% were already in stage IV and 18% in stage III (24). In the same way, we did not have sufficient data to describe the diagnostic/treatment pathway for early stages that underwent nonsurgical treatment. However, according to the available reports, only 1.8% of stages I and II NSCLC at diagnosis did not receive radical surgery as treatment in Tessin (26). Therefore, a definitive conclusion on the effectiveness of diagnostic and treatment pathways across urban–rural residences cannot be made. In the same way, we did not know if and how many patients were excluded from the surgical treatment because of delay. Further studies should be pursued in this sense.

We did not have a personal patients’ income; we used the insurance status as a surrogate. We did not consider other factors, such as the educational level, which might have influenced the outcome.

We retrospectively analyzed a prospectively collected database. The retrospective analysis carries an inherent bias, but it is necessary to critically evaluate the quality of the care. Furthermore, the study considered a limited number of patients compared to the published literature, but the use of a clinical database increased enormously the quality of data. Compared to epidemiological databases used for the vast majority of studies on this topic, there was no indirect extrapolation of data about patients’ characteristics or diagnostic steps.

## Conclusions

In conclusion, we pictured the real-world lung cancer surgical pathway at a local level. We identified significant differences in the timing of diagnosis and treatment between urban and rural populations, with the latter having a shorter route. Possible greater GP involvement might have contributed to this result, specifically in asymptomatic patients. Even though the results cannot be, and must not be, universally applied, our study gives some insights into a critical development of more equal and effective lung cancer pathways and suggests the need for a local tailored approach to lung cancer screening programs.

## Data Availability

The raw data supporting the conclusions of this article will be made available by the authors without undue reservation.
